# The Effect of Metformin on Adolescents with Type 1 Diabetes: A Systematic Review and Meta-Analysis of Randomized Controlled Trials

**DOI:** 10.1155/2016/3854071

**Published:** 2016-07-12

**Authors:** Wei Liu, Xiao-Jie Yang

**Affiliations:** ^1^Department of Cardiology, Guizhou Provincial People's Hospital, Guizhou 550002, China; ^2^Department of Endocrinology, Guizhou Provincial People's Hospital, Guizhou 550002, China

## Abstract

*Background*. The effect of metformin in combination with insulin in adolescents with type 1 diabetes (T1DM) is controversial.* Methods and Results*. The PubMed and EMBASE online databases were searched. Five double-blind randomized controlled trials (RCTs) that included 301 adolescents with T1DM were identified. Metformin plus insulin was associated with reduced hemoglobin A1C levels, total daily insulin dosage, body mass index (BMI), and body weight. However, the subgroup analysis demonstrated that HbA1c levels were not significantly changed in overweight/obese adolescents and were significantly reduced in the general patients. On the contrary, BMI and body weight were significantly reduced in overweight/obese adolescents but not in the general patients. Metformin was associated with higher incidence of adverse events.* Conclusions*. Among adolescents with T1DM, administering adjunctive metformin therapy in addition to insulin was associated with improved HbA1c levels, total daily insulin dosage, BMI, and body weight. However, there may be differences in the effects of this regimen between overweight/obese and nonobese adolescents. The risk of an adverse event may be increased with metformin treatment. These results provide strong evidence supporting future high-quality, large-sample trials.

## 1. Introduction

Type 1 diabetes mellitus (T1DM) is characterized by the immune-mediated depletion of *β*-cells, which results in deficient insulin secretion [1]. T1DM accounts for about 5% of all cases of diabetes, and three-quarters of all cases of T1DM are diagnosed in individuals <18 years old [2]. Early treatment is essential to prevent complications [3]. In addition to a deficiency in insulin secretion, insulin resistance is also considered to be a contributor in both normal-weight and overweight T1DM adolescents [4]. Insulin resistance makes glycemic control difficult [5, 6]. Additionally, insulin resistance has been demonstrated to increase cardiovascular disease risk factors in T1DM adolescents [7]. Intensive insulin treatment is required to achieve lower hemoglobin A1c (HbA1c) levels, which reduces microvascular and cardiovascular complications [8]. However, weight gain is a problem that is associated with intensive insulin treatment [9]. For adolescents with T1DM, weight gain has potentially serious metabolic consequences, including increased insulin resistance. Moreover, about 40% of adolescences with T1DM are overweight or obese [10].

Metformin is an oral antihyperglycemic drug that is commonly used to manage type 2 diabetes mellitus (T2DM) [11]. Its pharmacologic mechanisms include (1) reducing hepatic glucose production, (2) decreasing the intestinal absorption of glucose, and (3) improving insulin sensitivity by increasing peripheral glucose uptake and utilization [11]. Metformin was initially applied in adolescents with T1DM 40 years ago [12]. Several studies in recent years have evaluated the effects of administering metformin in combination with insulin in adolescents with T1DM [13–15]. However, the results of these studies have been contradictory and inconclusive regarding their effectiveness in glycemic control and changes in body weight and safety. Notably, the sample sizes in these studies are relatively small. One early review article focused on this issue [16], but it included only two studies containing 60 patients and was published 12 years ago [6, 17]. Therefore, we performed the current systematic review and meta-analysis to comprehensively evaluate the efficacy and safety of using metformin as an adjunct to insulin therapy in adolescents with T1DM.

## 2. Method

In the present study, we included trials that met the following criteria: (1) a randomized controlled trial (RCT) that used a placebo as the parallel; (2) trials that investigated the effect of using metformin as an adjunct to insulin therapy in adolescents with T1DM (age: less than 20 years); and (3) the duration of metformin therapy being 3 months or longer. Articles written in a non-English language were excluded. We also excluded publications in the following formats: abstracts, letters, editorials, and reviews.

### 2.1. The Search and Screening Strategies

The PubMed and EMBASE online databases (up to December 28, 2015) were searched to identify all publications related to metformin and T1DM. We also performed a manual search by scanning the references of the identified articles to find potentially relevant studies that were missed by the electronic searches.

Two authors (Wei Liu and Xiao-Jie Yang) independently screened all potentially relevant studies. A crude screening was performed at the level of title and abstract to briefly assess the study design (randomized trial versus open-label or other design) and to evaluate the effect of metformin on any results. If uncertainty existed, the full-text was examined to achieve a final decision. If there was a difference of opinion between the two screening authors, a discussion was used to resolve the issue. After the final screening, two authors (Wei Liu and Xiao-Jie Yang) independently extracted the data from all eligible articles. A prespecified table that contained the relevant items was used to help with data extraction.

### 2.2. Endpoints

The purpose of the present study was to assess the efficacy and safety of metformin in adolescents with T1DM. For efficacy endpoints, meta-analyses were performed to analyse changes in HbA1c levels, total daily insulin dosage, body mass index (BMI), and body weight from the baseline of the study to its completion between the metformin and placebo groups. Other clinical parameters were also summarily reviewed. The safety endpoints consisted of severe hypoglycemia, gastrointestinal events, and diabetic ketoacidosis. Both the summarized review table and the pooled forest plot are shown (Table 4 and Figures 6 and 7).

### 2.3. Assessment of Risk of Bias and Heterogeneity

The characteristics of the included studies were assessed using a combination of the Jadad scale and individual components that are known to affect estimates of intervention efficacy. The Jadad scale consists of three items pertaining to descriptions of randomization (0–2 points), double blinding (0–2 points), and dropouts and withdrawals (0-1 point) in a total of five scores, with a higher score indicating better quality [19]. High-quality trials were defined as those that scored higher than 2. Low-quality trials were defined as those that scored 2 or lower [19]. Additionally, the assessment to determine the concealment of allocation was made based on the criteria described by Schulz et al., in which the concealment of allocation was assessed as adequate, inadequate, or unclear [18].

Heterogeneity assessments were made by calculating the *I* ^2^ statistic, where *I* ^2^ < 50% was considered to indicate the heterogeneity might not be important, whereas *I* ^2^ > 50% indicated significant heterogeneity [20]. A random-effects model was used to pool the effect sizes in all of the analyses.

### 2.4. Subgroup and Sensitivity Analysis

We explored the impact of weight using a subgroup analysis. The trials were divided into overweight/obese and general subgroups. If heterogeneity was significant in the meta-analysis, a sensitivity analysis was performed by deselecting the studies one by one to determine the origin of the heterogeneity.

### 2.5. Statistical Analysis

All statistical analyses were performed in compliance with the statistical guidelines described in the* Cochrane Handbook for Systematic Reviews of Interventions Version 5.1.0 *[20]. We pooled the data into a forest plot if enough data were available and if the data were of sufficient quality. However, we only summarize the data if there was an insufficient amount or if its quality was too low to incorporate into the text or a table. For continuous data, mean differences (MD) were pooled to assess differences between groups under normal circumstances, while standardized mean differences (SMD) were used to assess data describing the same outcome that was obtained using a different method of measurement. Risk ratios (RRs) were calculated to evaluate differences in dichotomous data. All of these analyses were performed using RevMan software version 5.20 (The Nordic Cochrane Centre, Copenhagen, Denmark). A two-tailed *P* ≤ 0.05 was considered to indicate statistical significance.

The present systematic review and meta-analysis was performed in line with the recommendations of the PRISMA statement (Preferred Reporting Items for Systematic Reviews and Meta-Analyses) [21].

## 3. Results

### 3.1. Results of Search and Screening

**Figure 1:**
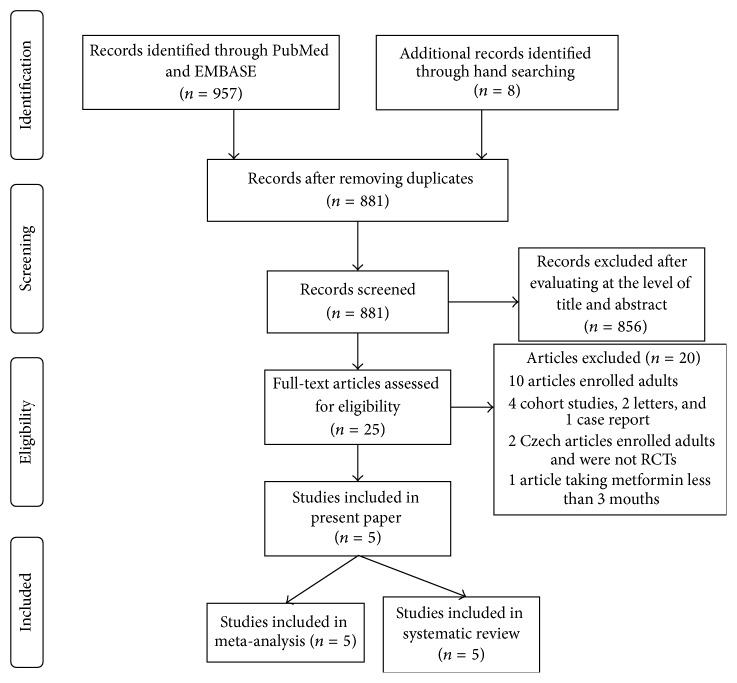
QUOROM (quality of reporting of meta-analyses) flow-chart of the study selection process.

An outline of the search and screening process used in this study is described in the QUOROM (quality of reporting of meta-analyses) flow-chart shown in Figure 1. A total of 965 relevant articles were identified. Of these, 957 were obtained from the PubMed and EMBASE databases, and the other 8 were obtained from a manual review of citations. We excluded the majority of articles after evaluating them individually at the level of title and abstract. Only 25 articles needed to be reevaluated using a full-text reading. After perusing the full-text of these 25 articles, 20 were excluded. Finally, the remaining 5 articles were included in the present systematically illustrated review and meta-analysis [6, 13–15, 17].

### 3.2. Characteristics of the Included Articles

The detailed characteristics of the included articles are shown in Table 1. All 5 of the articles were double-blind RCTs. A total of 301 adolescents were enrolled. The mean age of the patients was less than 20 years in all trials. The average duration of T1DM ranged from 5 to 10 years. The length of metformin therapy was 3 months or more, with the longest duration (9 months) in the study by Nwosu et al. [13]. The metformin dosages among the studies varied, with the total dosage varying from 1000 to 2000 mg per day. Of the included studies, 2 recruited only overweight/obese adolescents, and 3 enrolled general persons. In the present study, we performed subgroup analyses to evaluate the differences in the effects of metformin therapy between overweight/obese and general adolescents with T1DM.

### 3.3. The Quality of the Included Studies

Table 2 provides a detailed assessment of study quality. All five of the included studies were high-quality according to their Jadad score. Except for the study from Särnblad et al., which had a Jadad score of 3 [6], the other four studies each had a Jadad score of 5. Regarding the concealment of allocation, the most recent three studies, which were published in 2015, were adequate [13–15], while the results for the two studies published in 2003 were unclear [6, 17].

### 3.4. Glycemic Control and Insulin Dosage

**Figure 2:**
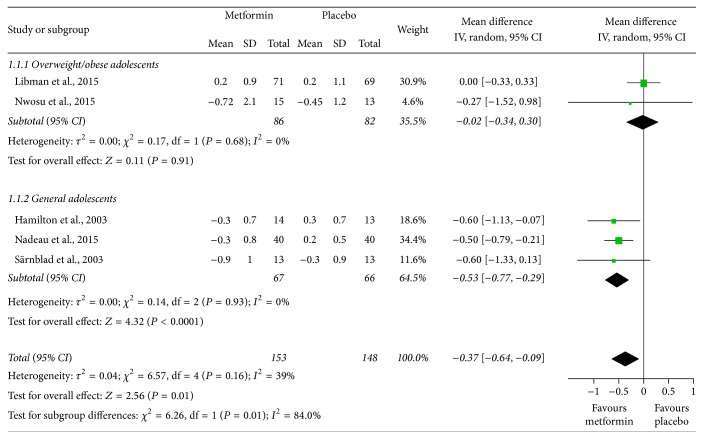
Mean differences in changes in hemoglobin A1c (HbA1c) levels from baseline to study termination between patients who received adjunctive metformin therapy or placebo in adolescents with type 1 diabetes mellitus. CI: confidence interval; IV: inverse variance.

**Figure 3:**
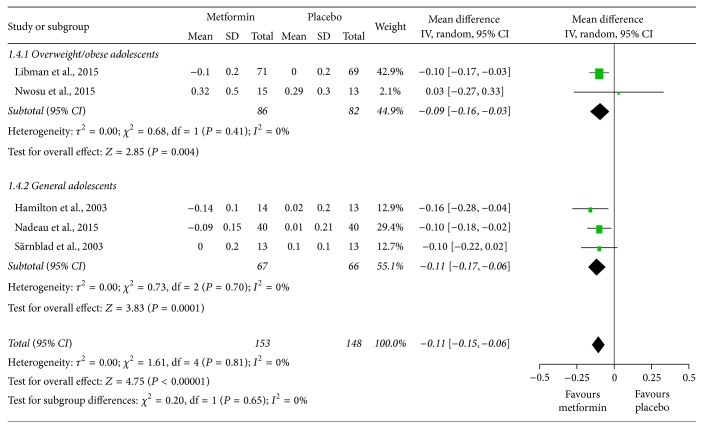
Mean differences in changes in total daily insulin dosage from baseline to study termination between patients who received adjunctive metformin therapy or placebo in adolescents with type 1 diabetes mellitus. CI: confidence interval; IV: inverse variance.

The pooled results showed that HbA1c levels (%) were slightly lower in the metformin therapy group than in the placebo-treated group (MD = −0.37, 95% confidence interval (CI): −0.64 to −0.09; Figure 2). However, the subgroup analysis showed that the effect might be different between overweight/obese and general adolescents (*I* ^2^ = 84%) and that HbA1c levels were not significantly altered in overweight/obese adolescents (MD = −0.02, 95% CI: −0.34 to 0.30), while they were significantly improved by 0.53% in the general group (MD = −0.53, 95% CI: −0.77 to −0.29).  Figure 3 shows that the total daily insulin dosage per kg of body weight (units/day/kg) was significantly lower in the groups treated with metformin in both overweight/obese and general adolescents (MD = −0.11, 95% CI: −0.15 to −0.06, and *I* ^2^ = 0%).

### 3.5. Change in Adiposity

**Figure 4:**
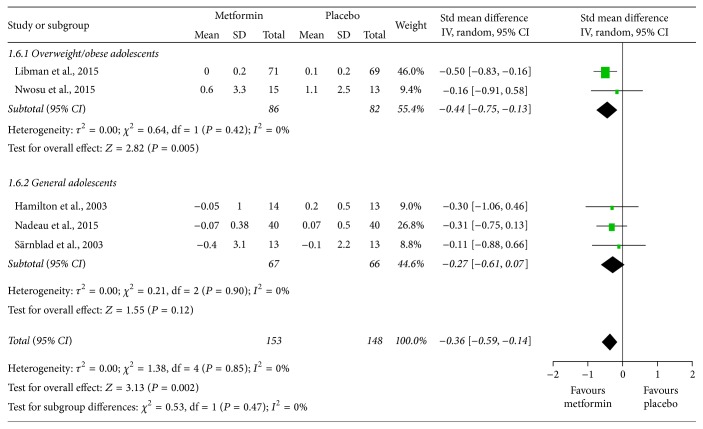
Standardized mean differences in changes in body mass index (BMI) from baseline to study termination between patients who received adjunctive metformin therapy or placebo in adolescents with type 1 diabetes mellitus. CI: confidence interval; IV: inverse variance.

**Figure 5:**
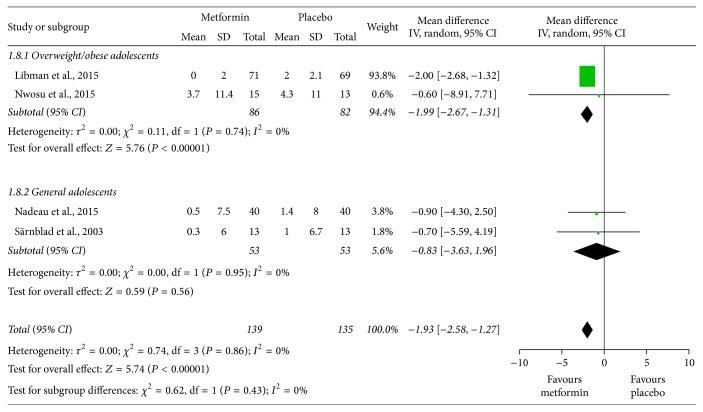
Mean differences in changes in body weight from baseline to study termination between patients who received adjunctive metformin therapy or placebo in adolescents with type 1 diabetes mellitus. CI: confidence interval; IV: inverse variance.

The meta-analyses indicated that metformin significantly decreased BMI (SMD = −0.36, 95% CI: −0.59 to −0.14; Figure 4) and body weight (MD = −1.93, 95% CI: −2.58 to −1.27; Figure 5) with no important interstudy heterogeneity (*I* ^2^ = 0% in both meta-analyses). Intriguingly, the subgroup analyses showed that BMI and body weight were significantly reduced in overweight/obese adolescents, while in the general participants, BMI and body weight did not significantly change but trended towards reduction.

### 3.6. Adverse Events

**Figure 6:**
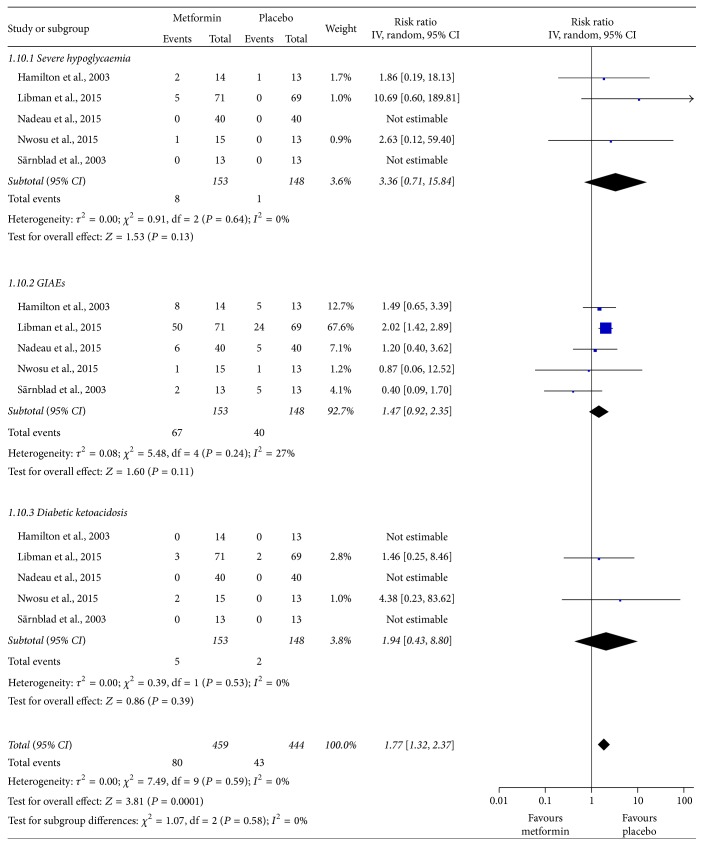
Meta-analysis of adverse events between patients who received adjunctive metformin therapy or placebo in adolescents with type 1 diabetes mellitus. CI: confidence interval; GIAEs: gastrointestinal adverse events; IV: inverse variance.

**Figure 7:**
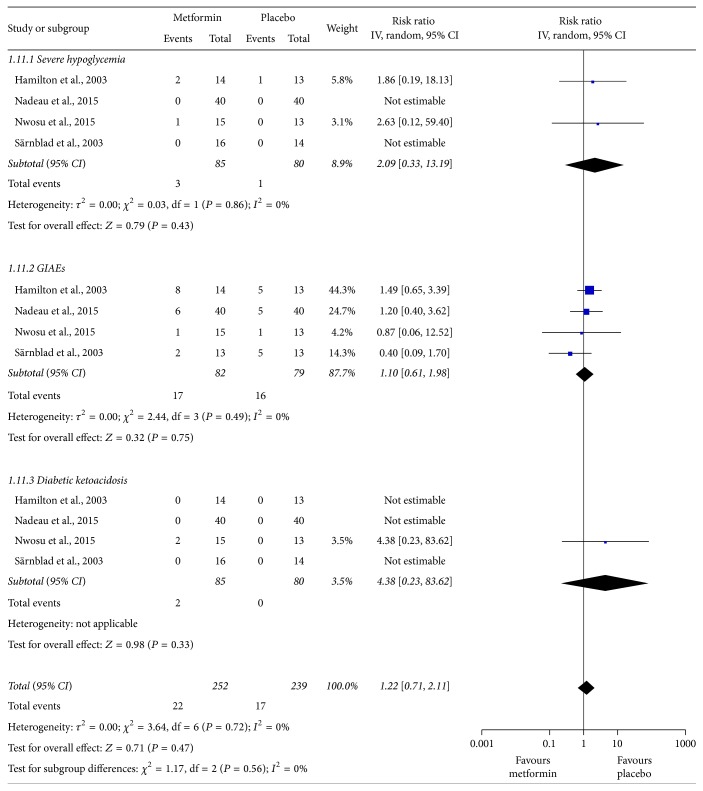
The sensitivity analysis of adverse events (after the study from Libman et al. was omitted) between adjunctive metformin therapy and placebo in adolescents with type 1 diabetes mellitus. CI: confidence interval; GIAEs: gastrointestinal adverse events; IV: inverse variance.

We have summarized the observed adverse events in Table 3. Gastrointestinal discomfort was the most common adverse event. The incidence of gastrointestinal adverse events varied from 6.7 to 70.4% in different studies, with an average rate of 43.8% in the metformin group and 27.0% in the placebo group. The occurrence of other adverse events was relatively lower. There was a trend towards a higher incidence of severe hypoglycemia in patients who received metformin therapy. For a more visual comparison of these adverse events, we have presented the results of the meta-analysis. The pooled results indicated that metformin therapy might increase the total incidence of adverse events in adolescents with T1DM by 1.77-fold (RR = 1.77, 95% CI: 1.32 to 2.37, and *I* ^2^ = 0%; Figure 6). A sensitivity analysis showed that this result was distinctly affected by a study by Libman et al. [15]. After omitting this study, adverse events were no longer significantly increased in the metformin group (Figure 7).

### 3.7. Other Clinical Parameters

Some studies reported a variety of other clinical parameters. We have systematically summarized these outcomes in Table 4. The results suggest that metformin therapy may have no significant effect on lipid parameters, blood pressure, or metabolic effects. How much insulin sensitivity, which was measured using different methods, changed was controversial across these studies. Insulin sensitivity was not significantly altered in the studies by Nwosu et al. [13]and Hamilton et al. [17] but was slightly improved in the study by Särnblad et al. [6].

## 4. Discussion

The present systematic review and meta-analysis analysed the data from five high-quality double-blind RCTs to evaluate the effect of metformin on adolescents with T1DM. The pooled outcomes demonstrated that (1) HbA1c levels were slightly better (by 0.37%) in the metformin group than in the placebo group, but the subgroup analysis indicated that this improvement could be attributed to the studies that included general adolescents instead of only overweight/obese individuals; (2) administering metformin as an adjunct therapy significantly decreased BMI and body weight, and its effectiveness was more remarkable in overweight/obese adolescents; (3) metformin therapy was associated with a significant reduction in the total daily insulin dosage in both overweight/obese and general adolescents with T1DM; and (4) treating adolescents with T1DM with metformin may be associated with an increase in adverse events.

Glycemic control is vital in patients with diabetes. Reducing HbA1c levels decreases the incidence of microvascular and macrovascular complications in T1DM. The American Diabetes Association (ADA) recommends an HbA1c goal of <7.5% across all pediatric age-groups [22]. In the present study, we found that there was a 0.37% decline in HbA1c levels in the adolescent patients with T1DM who received adjunctive metformin therapy. Moreover, we found that metformin therapy was associated with an approximately 0.53% reduction in HbA1c levels in general adolescents but that there was no difference of HbA1c levels in overweight/obese adolescents between metformin therapy and placebo. Many studies have demonstrated that the glycemic response to metformin in nonobese and overweight/obese patients is similar to the response in T2DM [23–25]. However, how the composition of the body affects the glycemic response to metformin has not been well studied in T1DM. Although our results are based on data obtained from five high-quality double-blind RCTs and although we observed no significant heterogeneity, interpretations of these results still should be made cautiously, given the small sample size and short-term therapeutic duration.

Two previous meta-analyses that included both adults and adolescents with T1DM found that metformin had no impact on HbA1c levels and that adults with T1DM showed barely any glycemic response to metformin [26, 27]. Therefore, there may be differences in the glycemic response to metformin between adolescents and adults with T1DM. Future studies in this field should explore and better define these differences.

Improved insulin sensitivity is one of the pharmacological mechanisms by which metformin functions. Our meta-analysis demonstrated that there was a significant reduction in the total daily insulin dosage when adjunctive metformin was administered in adolescents with T1DM. Moreover, in our meta-analysis, we also observed a reduction in BMI and body weight, and decreases in BMI and body weight might further improve insulin sensitivity. Moreover, our results show that BMI was not significantly reduced in general adolescents, a result that was similar to the results of other studies involving T2DM patients [28]. This partially abolishes the concern that excessive weight loss occurs in normal-weight metformin users.

In terms of adverse events, there was obvious heterogeneity among the studies in their definitions, degree of supervision, and duration of follow-up. Although the four relatively small trials did not reach statistical significance for any adverse events, the largest trial, by Libman et al., did find that the rate of metformin-associated gastrointestinal events was increased [15]. However, the largest trial also reported that no system or organ class other than the gastrointestinal system showed a significant treatment-related group difference [15]. The pooled safety outcome data revealed that metformin was associated with a 1.77-fold increase in the total rate of adverse events. The sensitivity analysis showed that the study by Libman et al. was the origin of heterogeneity [15]. In that study, 2000 mg of metformin was taken per day, and more gastrointestinal events and severe hypoglycemia were reported [15]. In the instructions for administering metformin, a maximum daily dose of 2000 mg is recommended in pediatric patients (10–16 years of age) [11]. Therefore, the daily dose in the study by Libman et al. might actually have been an overdose. A smaller daily dose, such as 500 mg twice daily, would probably lessen gastrointestinal events and severe hypoglycemia. Moreover, the strict monitoring for adverse events in this study may have led to the discovery of more cases of gastrointestinal discomfort than were identified in the other studies. However, although many gastrointestinal events were reported, few of the subjects dropped out for this reason. It is therefore plausible that metformin can be tolerated in most patients with T1DM. In spite of this, we should pay attention to metformin-associated adverse events, such as hypoglycemia and gastrointestinal discomfort, according to the reported experiences of T2DM patients who undergo these treatments.

None of the included studies reported cardiovascular events. The underlying cause of the cardiovascular protective effect that is provided by metformin in patients with T1DM remains unknown. We reviewed limited data in adolescents with T1DM and did not find that metformin significantly improved risk factors related to cardiovascular disease, such as blood pressure and lipid levels, despite the fact that some of the included studies observed that metformin significantly decreased lipid levels in adults with T1DM. However, we did observe improvements in HbA1c levels, BMI, and the total insulin daily dosage in the present study. These factors have an impact on reducing cardiovascular disease. The ongoing EMERALD (Effects of Metformin on Cardiovascular Function in Adolescents with Type 1 Diabetes) trial (NCT01808690) may provide additional useful information that can address this effect [29].

## 5. Limitations

The present systematic review and meta-analysis has some limitations. First, five eligible studies were identified, and they contained only 301 T1DM adolescents. The sample size was therefore small, and the therapeutic duration for metformin was relatively short in these studies. We certainly acknowledge that this is a limitation, but the outcomes are unlikely to be significantly altered in the future given that all of these trials were double-blind, high-quality RCTs. Second, we excluded non-English studies, which might have resulted in publication bias. However, the great majority of high-quality trials are published in English or at least contain an English abstract. We found only two relevant studies that were published in Czech [30, 31]. However, except for their language, the design and type of enrolled adults in these studies were not aligned with our inclusion criteria. Therefore, neither study was excluded because of the language of the abstracts.

In summary, this systematic review and meta-analysis of double-blind RCTs demonstrates that administering adjunctive metformin therapy in adolescents with T1DM is associated with a mild improvement in HbA1c levels and small reductions in BMI and body weight, but these effects might differ between overweight/obese and nonobese adolescents. In addition, the total daily insulin dosage requirement was observed to be lower in treated patients, which may indicate improved insulin sensitivity. It is not possible to achieve a final judgment on the questions put forward in this analysis from our outcomes alone. Further studies that are high-quality and that include a large sample size and long-term follow-up, especially studies with hard endpoints, are needed to guide the future use of metformin as a therapy in adolescents with T1DM.

## Figures and Tables

**Table 1:** Study design and baseline characteristics of the included studies.

Study and publication year	Study design	T1DM duration, y		Sample size		Male		Age, y		BMI, kg/m^2^		HbA1c, %		Duration of therapy	Final dose of metformin per day	Target participants
		Met	PL	Met	PL	Met	PL	Met	PL	Met	PL	Met	PL			
Libman et al., 2015 [15]	Multicenter, double-blind RCT	7.5 (3.6)	6.4 (3.0)	71	69	27	21	15.4 (1.7)	15.1 (1.8)	1.6^#^ (0.4)	1.7^#^ (0.3)	8.8 (0.8)	8.8 (0.7)	26 weeks	2000 mg	Overweight/obese adolescents
Nwosu et al., 2015 [13]	Single-center, double-blind RCT	5.7 (4.4)	5.7 (5.0)	15	13	8	5	15.0 (2.5)	14.5 (3.1)	28.0 (5.4)	27.7 (4.1)	9.3 (1.5)	8.7 (0.4)	9 months	1000 mg	Overweight/obese adolescents
Nadeau et al., 2015 [14]	Single-center, double-blind RCT	6.7 (3.6)	6.3 (3.5)	40	40	59% males in total		15.9 (1.7)	16.0 (1.6)	23.5 (3.0)	24.3 (4.1)	9.5 (1.3)	9.4 (1.1)	6 months	1000 mg	General adolescents
Särnblad et al., 2003 [6]	Multicenter, double-blind RCT	9.1 (5.0)	7.1 (3.0)	13	13	5	4	17.2 (1.7)	16.9 (1.4)	23.5^*∗*^ (18.6–35.4)	23.9^*∗*^ (17–29.2)	9.6 (1.0)	9.5 (1.2)	3 mouths	2000 mg	General adolescents
Hamilton et al., 2003 [17]	Single-center, double-blind RCT	9.9 (4.4)	7.0 (3.8)	14	13	6	7	15.9 (1.9)	16.0 (1.7)	22.8 (4.2)	25.7 (2.9)	9.3 (1.4)	8.6 (0.8)	3 months	1000 mg, 1500 mg, or 2000 mg according to body weight	General adolescents

^#^BMI was calculated using height and weight and adjusted for age and sex using growth chart tables obtained from the centers for disease control and prevention. BMI *Z*-scores were used in this study. ^*∗*^The data are shown as the median (range).

Data are shown as numbers or means (SD) unless otherwise indicated. RCT: randomized controlled trial; T1DM: type 1 diabetes mellitus; HbA1c: hemoglobin A1c; BMI: body mass index; Met: metformin; PL: placebo.

**Table 2:** Quality assessment of individual studies.

Study and publication year	Randomization	Double blinding	Dropouts	Jadad score^*∗*^	Concealment of allocation^#^
Libman et al., 2015 [15]	2	2	1	5	Adequate
Nwosu et al., 2015 [13]	2	2	1	5	Adequate
Nadeau et al., 2015 [14]	2	2	1	5	Adequate
Särnblad et al., 2003 [6]	1	1	1	3	Unclear
Hamilton et al., 2003 [17]	2	2	1	5	Unclear

^*∗*^The Jadad scale consists of three items related to descriptions of randomization (0–2 points), double blinding (0–2 points), and dropouts and withdrawals (0-1 point) for a total of five scores. Higher scores indicate better quality. High-quality trials were defined as those that scored more than 2. Low-quality trials were defined as those that scored 2 or less.

^#^Concealment of allocation was assessed as adequate, inadequate, or unclear based on the criteria described by Schulz et al. [18].

**Table 3:** Summary of adverse events.

Study and publication year	Sample size		Severe hypoglycemia		Gastrointestinal AEs		Diabetic ketoacidosis	
	Met	PL	Met	PL	Met	PL	Met	PL
Libman et al., 2015 [15]	71	69	5 (7.0%)	0 (0%)	50 (70.4%)	24 (34.8%)	3 (4.2%)	2 (2.9%)
Nwosu et al., 2015 [13]	15	13	1 (6.7%)	0 (0%)	1 (6.7%)	1 (6.7%)	2 (13.3%)	0 (0%)
Nadeau et al., 2015 [14]	40	40	0 (0%)	0 (0%)	6 (15.0%)	5 (12.5%)	0 (0%)	0 (0%)
Särnblad et al., 2003 [6]	13	13	0 (0%)	0 (0%)	2 (15.4%)	5 (38.5%)	0 (0%)	0 (0%)
Hamilton et al., 2003 [17]	14	13	2 (14.3%)	1 (7.7%)	8 (57.1%)	5 (38.5%)	0 (0%)	0 (0%)

Total	153	148	8 (5.2%)	1 (0.7%)	67 (43.8%)	40 (27.0%)	5 (3.3%)	2 (1.4%)

The data are shown as numbers (%). Met: metformin; PL: placebo.

**Table 4:** Summary of other clinical parameters.

Study and publication year	Other outcomes
Libman et al., 2015 [15]	No significant differences were observed between groups in percentile changes in blood pressure or the levels of lipids, inflammatory markers, C-reactive protein, or C peptide.

Nwosu et al., 2015 [13]	There were no significant differences between the groups in changes in blood pressure, the level of insulin sensitivity factor (ISF), ideal blood glucose (IBG), or insulin-to-carbohydrate ratio (ICR).

Nadeau et al., 2015 [14]	No significant differences were observed in changes in blood pressure or lipid levels between groups.

Särnblad et al., 2003 [6]	Peripheral insulin sensitivity was increased in the group that received metformin. No differences were observed in the levels of blood lipids, insulin-like growth factor I (IGF-I), or IGF-binding protein-1 (IGFBP-1) between the two groups.

Hamilton et al., 2003 [17]	No changes in insulin sensitivity were observed according to the frequently sampled intravenous glucose tolerance test (FSIGT). Cholesterol and triglyceride levels did not change during the study period.
